# Functional Laterality of Task-Evoked Activation in Sensorimotor Cortex of Preterm Infants: An Optimized 3 T fMRI Study Employing a Customized Neonatal Head Coil

**DOI:** 10.1371/journal.pone.0169392

**Published:** 2017-01-11

**Authors:** Lukas Scheef, Jurek A. Nordmeyer-Massner, Adam PR Smith-Collins, Nicole Müller, Gaby Stegmann-Woessner, Jacob Jankowski, Jürgen Gieseke, Mark Born, Hermann Seitz, Peter Bartmann, Hans H. Schild, Klaas P. Pruessmann, Axel Heep, Henning Boecker

**Affiliations:** 1 Department of Radiology, University of Bonn, Bonn, Germany; 2 Institute for Biomedical Engineering, University of Zurich and ETH Zurich, Zurich, Switzerland; 3 School of Clinical Sciences, University of Bristol, Bristol, United Kingdom; 4 Department of Neonatology, University of Bonn, Bonn, Germany; 5 Philips Medical Systems, Hamburg, Germany; 6 Department of Mechanical Engineering and Marine Technology, University of Rostock, Rostock, Germany; Australian Research Council Centre of Excellence in Cognition and its Disorders, AUSTRALIA

## Abstract

**Background:**

Functional magnetic resonance imaging (fMRI) in neonates has been introduced as a non-invasive method for studying sensorimotor processing in the developing brain. However, previous neonatal studies have delivered conflicting results regarding localization, lateralization, and directionality of blood oxygenation level dependent (BOLD) responses in sensorimotor cortex (SMC). Amongst the confounding factors in interpreting neonatal fMRI studies include the use of standard adult MR-coils providing insufficient signal to noise, and liberal statistical thresholds, compromising clinical interpretation at the single subject level.

**Patients / methods:**

Here, we employed a custom-designed neonatal MR-coil adapted and optimized to the head size of a newborn in order to improve robustness, reliability and validity of neonatal sensorimotor fMRI.

Thirteen preterm infants with a median gestational age of 26 weeks were scanned at term-corrected age using a prototype 8-channel neonatal head coil at 3T (Achieva, Philips, Best, NL). Sensorimotor stimulation was elicited by passive extension/flexion of the elbow at 1 Hz in a block design. Analysis of temporal signal to noise ratio (tSNR) was performed on the whole brain and the SMC, and was compared to data acquired with an ‘adult’ 8 channel head coil published previously. Task-evoked activation was determined by single-subject SPM8 analyses, thresholded at p < 0.05, whole-brain FWE-corrected.

**Results:**

Using a custom-designed neonatal MR-coil, we found significant positive BOLD responses in contralateral SMC after unilateral passive sensorimotor stimulation in all neonates (analyses restricted to artifact-free data sets = 8/13). Improved imaging characteristics of the neonatal MR-coil were evidenced by additional phantom and in vivo tSNR measurements: phantom studies revealed a 240% global increase in tSNR; in vivo studies revealed a 73% global and a 55% local (SMC) increase in tSNR, as compared to the ‘adult’ MR-coil.

**Conclusions:**

Our findings strengthen the importance of using optimized coil settings for neonatal fMRI, yielding robust and reproducible SMC activation at the single subject level. We conclude that functional lateralization of SMC activation, as found in children and adults, is already present in the newborn period.

## Introduction

Previous studies have demonstrated that the development of subcortico-cortical connections is disrupted following preterm birth [1], in turn causing adverse neurodevelopmental outcomes across multiple cognitive domains [2–6]. Prematurity is also associated with differing degrees of motor impairment that range from manifest cerebral palsy (CP) to a spectrum of more subtle motor skill impairments that belong to the frequent "hidden disabilities" in those born preterm [7], which can be identified in approximately 40% of former preterm adults [8]. Clinical diagnosis of delayed or impaired motor development and function is challenging in early infancy, and is usually not recognized earlier than 6–10 months of age [9]. Thus, methods that allow early prediction of adverse motor outcome have fundamental clinical impact. Both cranial ultrasound (cUS) and magnetic resonance imaging (MRI) have value in predicting motor outcomes. MRI outperforms cUS in detecting subtle white matter injury at term-equivalent age (TEA) [10], with higher predictive value associated with detection of parenchymal lesions than abnormalities of delayed myelination [11]. In preterm neonates, white matter abnormalities (on structural MRI) predict later microstructural disorganization (on diffusion MRI) of the corticospinal tract (CST) by the age of 7 years [12]. For early clinical counseling, however, structural measures currently have limited value for predicting the development of CP: while the negative predictive value of a normal structural MRI at TEA approaches 100% for predicting CP at the age of five years, the positive predictive value of multiple major structural lesions reaches only 44% [13]. Likewise, the sensitivity of sequential postnatal cUS for predicting CP reaches only 46% [14], although other studies have reported better predictive accuracies of about 60% [15]. Direct comparisons of the two methods in very low birth weight (VLBW) preterm infants revealed that MRI outperforms cUS in the specificity of predicting neuromotor outcomes tested until 18 months of corrected age [11]. Parenchymal lesions on MRI predict cerebral palsy with a sensitivity of 82%, as compared to 58% for cUS.

While structural brain imaging has prognostic value, even the best reported predictors fall short of ideal characteristics in terms of positive and negative predictive value. Over recent years, a number of researchers have investigated whether early investigation of neonatal sensorimotor function using functional magnetic resonance imaging (fMRI) at TEA is sensitive and specific in detecting relevant functional impairments of the CST (particularly when structural white matter lesions are present), potentially serving as a predictor for long-term motor outcomes in at-risk neonates. If functional alterations associated with clinically salient CST lesions were identifiable using neonatal fMRI, this would be a major breakthrough establishing a path for early and focused physiotherapy with the aim of exploiting the full range of cortical plasticity characteristic of this early stage of brain development.

Amongst the plethora of available methods (ranging from early and longitudinal clinical assessments, cranial ultrasound, structural and diffusion MRI), functional assessment provided by task-based fMRI is unique, as it allows challenging sensorimotor function at the systems level *in vivo*. Neonatal sensorimotor fMRI research has focused primarily on the detection of BOLD signal changes in the primary sensorimotor cortex (SMC). The SMC constitutes the classical motor executive region and the cerebral “origin” of the CST, which closely interacts with sensory input conveyed to the primary sensory cortex. The contralateral SMC (i.e. the pre- and postcentral gyrus) is consistently activated by unilateral active or passive limb movements in fMRI studies, both in children and adults [16]. So far, neonatal fMRI studies investigating the sensorimotor system have employed mostly passive sensorimotor stimulation paradigms, ranging from manually applied forearm traction [17], use of manually inflatable [18, 19] or computer programmable balloons [20, 21], to fMRI-compatible robotic devices [22, 23]. In addition, a method has been recently introduced to measure spontaneous neonatal wrist movements [24]. Evoked BOLD responses in the SMC can be recorded using block- [17–20] or event-related [21, 24] fMRI study designs. Despite consistent involvement of the SMC, studies have so far yielded conflicting results, some identifying predominantly positive [20, 21, 24], others negative [17], or mixed positive/negative [19] BOLD signal responses in the SMC. More recently, age-related HRF-modelling of event-related fMRI data has improved the specificity of neonatal fMRI [25], demonstrating predominantly lateralized positive BOLD responses in the SMC [21] at early ages (30 to 43 post-conceptual weeks) [24]. These recent observations challenge older findings, which have reported more bilaterally distributed or predominantly negative task-evoked BOLD responses in the SMC. These differences raise questions about the validity of some previous findings, in particular in studies hampered by poor signal or application of liberal statistical criteria with the risk of reporting false-positive findings.

Interestingly, the novel findings based on age-related HRF-modelling suggest that the neonatal pattern of activation already resembles the prototypical activation pattern observed in healthy older children that activate predominantly the contralateral SMC after unilateral limb movements [16], similar to adults [26–28]. The possibility of detecting mature functional SMC responses in neonates is consistent with anatomical findings, indicating that human corticospinal connections are detectable as early as 24 weeks post-conceptional age (PCA) up to the level of the lower cervical spinal cord. This is followed by more extensive innervation of spinal neurons (including motor neurons) and myelination of corticospinal axons beginning during prenatal development [29]. Within the first two postnatal years, bilateral corticospinal connections undergo lateralization through successive elimination of most ipsilateral connections [30–32].

From a clinical perspective reliable characterization of SMC activations in response to sensorimotor stimulation at the single-subject level is a key target. Developing a suitable and robust method for characterizing SMC activity at high sensitivity, specificity, and reproducibility would provide a basis for detecting aberrant SMC activation in affected individuals. Whilst improved HRF-modelling of data after acquisition may contribute to this, another important element is enhancing signal quality, and opportunity afforded by recent advances in developing dedicated neonatal head coils. Adaptation of neonatal fMRI setting providing head coils adjusted to the neonatal head geometry and size, as well as an increasing number of coil elements, will improve the quality of MRI data [33–35]. A recent study utilizing a customized head coil with 32 channels for infants (0.5 to 4 years of age) demonstrated up to a 3.6-fold increase in signal-to-noise ratio (SNR), with improved geometry of coil elements also enabling accelerated imaging, compared to 32-channel adult coils [36].

Commonly, neonatal fMRI studies have, however, been conducted using standard equipment designed for adult heads [17] or best-fit approximations like knee coils [37]. While work from Erberich et al. has also reported neonatal fMRI using “neonatal” sized head coils integrated into neonatal MRI-compatible incubators at 1.5 T [18, 19], more recently, integration of 32 channel receive array coils into a dedicated neonatal brain imaging system at 3T has been implemented [38]. In this study we used a custom-designed 8-Channel head coil that was build and optimized to cover the neonatal head. We investigated the robustness and reproducibility of BOLD responses in the SMC of preterm infants at TEA in response to unilateral passive sensorimotor stimulation. Exploiting the optimized signal-to-noise ratio (SNR) of this dedicated head coil, we aimed to study whether SMC activity could be reproducibly identified in neonates at the single-subject level using standard analysis procedures and applying stringent statistical criteria (p < 0.05, whole brain FWE-corrected). We hypothesized, based on the recent literature, that the use of a customized neonatal head coil would yield robust and reproducible contralateral positive BOLD responses in the SMC. Furthermore, we characterize coil performance by phantom measurements and to compare the fMRI results to data acquired with a standardized 8-channel adult head coil [17].

## Methods

### Study design / patients

This prospective cohort study recruited 13 preterm infants, median gestational age 26 1/7 weeks (range 22 6/7–28 0/7 weeks) who were admitted to the tertiary neonatal intensive care unit at the University of Bonn between July 2010 and May 2013. The fMRI was performed as an adjunct to a clinical MRI protocol at term equivalent age (median 39 weeks + 2 days post-conceptional age (PCA); range 37+6–42+6 weeks/days). The structural/clinical MRI was successful in all study patients (n = 13).

In 8/13 infants, the fMRI protocol was successfully completed: Three data sets had to be excluded from analysis due to hardware failure related poor data quality, two data sets had to be withdrawn from further analysis due to excessive task-associated head movement. In two of the eight remaining neonates, we were able to perform two successive fMRI runs: a) stimulation of the right arm followed by stimulation of the left arm (NeoNat-06), b) stimulation of the right arm followed by stimulation of the right arm (NeoNat-11). In all other patients additional fMRI runs could not be completed due to excessive movement artifacts (waking up during the session).

Study inclusion/exclusion criteria, infant pre-scanning assessment, monitoring during MRI, acoustic protection and pre/post MRI Brainstem Evoked Response Audiometry studies were as previously reported [17]. A standardized neurological newborn examination as well as an assessment of psychomotor development at the age of 6–24 months PCA [39] was performed in all study infants. Serial cranial ultrasound imaging (cUS) was performed as part of clinical routine in study infants as previously described [17].

Perinatal brain damage was characterized according to the degree of intraventricular hemorrhage (IVH), white matter damage (WMD) and ventricular dilatation (VD) diagnosed on serial cUS investigations and on conventional MRI (see below) at term-equivalent age: cUS was performed using an 8.5–10 MHz transducer (Vingmed Vivid FiVe) after birth, at days 7 and 28 postnatal age and at term-equivalent age. The sonographic findings of IVH and WMD were classified using the criteria of Papile [40].: a) Grade I (mild)–germinal matrix hemorrhage with no or minimal intraventricular hemorrhage; b) Grade II (moderate)—intraventricular hemorrhage (10%-50% of ventricular area in parasagittal scan); c) Grade III (severe)—(>50% of ventricular area in parasagittal scan). d) Grade IV—apparent periventricular hemorrhagic infarction. WMD was defined as single or multiple cystic periventricular leukomalacia or gross cystic white matter defect after hemorrhagic infarction of the periventricular white matter.

The study protocol was approved by the Committee on the Ethics of the Medical Faculty of the University of Bonn, Germany (www.ethik.meb.uni-bonn.de; Permit Number: 018–06). In all investigated infants, written parental consent was obtained. This study was carried out in strict accordance with the declaration of Helsinki (current revision).

### Development of a head model and a custom-designed 8-channel head coil

**Fig 1:**
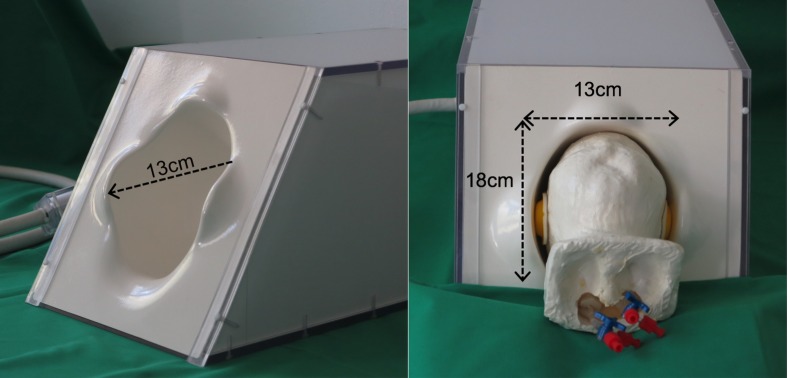
Image of the custom 8-channel head coil. The head model, representing the average head shape of a 6-month-old infant, which was used to optimize the coil shape, is inserted. Please note, that the model head fits closely into the coil when the (yellow) mini muff acoustic shells are applied. The extension of the moulded coil shell in x / y / z directions are 13 cm / 18 cm / 13 cm.

For MR imaging a custom-designed 8-channel head coil was developed that is optimized for the shape and size of the infant head. For this purpose a head-model based on 23 T1-weighted MRI datasets of term-born babies scanned at the age of 6 months was constructed (**Fig 1**). This age was chosen in order to increase the applicability of the coil, i.e. to perform longitudinal studies. A 3D surface file is provided in th**e S1 File**. The T1-data sets were rigidly co-registered using SPM8 (Wellcome Department of Imaging Neuroscience, London, United Kingdom) based on Matlab (The Mathworks Inc., Natick, MA). The outer scalp surface was extracted using BETSURF, a part of the functional software library (FSL: [41], BET: [42]. The resulting binary masks were added, thresholded at 95% and smoothed (smoothing kernel: 8x8x8mm^3^) to obtain a smooth representation of the head shape (SPM8). The contour files of the inner and outer skull surface were imported into in the machine software Rapix3D (Forwiss, Passau, Germany) that controls the 3D printing process. The head model was then built using a 3D printer type VX500 (Voxeljet Technology GmbH, Augsburg, Germany) using the powder material Solupor (Voxeljet, Augsburg, Germany). Thereafter, the parts were removed from the job box, cleaned with an air blower, and dried for 24 h in an oven at 40° C [43].

The neonatal coil consists of seven coil elements placed around the circumference of the head with an additional circular coil element on top. The coil elements were mounted on a glass fiber enforced moulded shell. The size and overlap of the coil elements was chosen to make use of geometric decoupling. In addition, pre-amplifier decoupling was used. The shell was tilted by approximately 45° to optimize the brain coverage when patients are placed in supine position inside the MR scanner. As outlined in **Fig 1**, the x / y / z dimensions of the neonatal coil are 13 cm / 18 cm / 13 cm. Following technical assessment and appropriate safety testing certification (Philips Healthcare, Best, NL) this prototype coil was used for structural and functional measurements.

### Structural MRI

Structural MRI was performed on a clinical MRI system (Philips Achieva 3 T MRI, Best, NL), equipped with the custom built 8-channel head coil described above. Infants were fed orally to promote natural sleep; additionally, chloral hydrate (50mg/kg) was administered via a gastric tube for sedation 5–10 minutes prior to MRI. Vital signs (body temperature, heart rate and oxygen saturation) of the infants studied were continuously monitored during the whole MRI procedure (FTI-10, FISO Technologies Inc. Québec, Canada; Nonin 8600 F0, Nonin Medical Inc., Minnesota, USA). A triple acoustic protection (ear prongs, mini muff acoustic shells, and Philips Noise Reduction Acoustic Hood System) was used for all infants. To rule out any relevant hearing impairment associated with the MRI, brainstem evoked response audiometry (BERA; Echo-screen system; Mack Medizintechnik, Pfaffenhofen, Germany) was performed pre/post MRI.

The structural imaging protocol consisted of a 3D-T1 Turbo Field Echo TFE sequence (TE/TR/FLIP: 4.6 msec / 9.7 msec / 8°, Turbo Factor 65, no averages, FOV: 150 x 150 x 150 mm^3^, scan percentage: 78%, reconstructed resolution: 0.94 x 0.94 x 0.94 mm^3^, scan duration ~ 5 min) and a high resolution 3D-T2 TSE sequence (TE/TR/FLIP: 362 msec / 2500 msec / 90°, Turbo Factor 115, 3 averages, FOV: 142.5 x 180 x 94 mm^3^, scan percentage: 100%, reconstructed resolution: 0.8 x 0.8 x 0.8 mm^3^, scan duration ~ 8 min).

### Functional imaging of passive sensorimotor stimulation

The fMRI was performed following the structural MRI sequences. To be able to compare the acquired data with published data and aiming to study individually reproducible SMC activation, we attempted to run two subsequent fMRI sequences in each patient. The fMRI sequences were different in chosen echo time (i) TE 35 msec (ii) TE 40 msec. The protocol for the first sequence (i) was as follows: single shot EPI, TE/TR/flip: 35 msec / 2400 msec / 90°, SENSE-factor 2, spatial resolution: 3 x 3 x 3.6 mm^3^, 250 volumes/scan, scan duration: 10.5 min). The second sequence (ii) was acquired with a higher spatial resolution (2 x 2 x 2.5 mm^3^) and TE 40ms, all other imaging parameters were kept constant.

Passive sensorimotor stimulation containing of unilateral elbow extension/flexion movements was delivered in a block-like fashion as previously described [17]. The paradigm consisted of alternating 10 periods of rest (no movement, 30 seconds each) and 10 periods of activation (passive movement, 30 seconds each).

### Data preprocessing and statistical analysis

Preprocessing and data analysis was performed with SPM8 (Wellcome Trust Centre for Neuroimaging, London, United Kingdom). All functional images were spatially realigned to the first functional image to reduce head motion during fMRI, and corrected for slice acquisition time differences. The resulting data sets were co-registered to the individual T2-weighted MRI and normalized to the T2-GA-39 template of the Imperial College high-definition spatio-temporal neonatal brain atlas [44]. This was accomplished by applying the normalization transformation obtained for the T2-weighted structural data set to the pre-processed functional data set. For individual analysis the data were initially spatially smoothed using a 6 mm isotropic Gaussian kernel.

In order to detect artifacts, abrupt head motions, intensity drifts etc., each pre-processed data set was averaged across time on a voxel-by-voxel basis and subtracted from each volume of the time series. In the same way as the mean volume, a standard deviation volume was calculated. All volumes of a series deviating more than 2 standard deviations from the series mean when averaging across all brain voxels were identified. This information was later used to set up a regressor to account for these volumes in the model (see below). A complete data set was excluded from the analysis if either excessive head motion was recognized during scanning or if any of the motion parameters were significantly correlated with the stimulation paradigm (p < 0.001) on later data analysis.

The data were modeled using the principles of the general linear model. The design matrix consisted of 2 event types (movement and rest) modeled using a square wave function convolved with the canonical adult hemodynamic response function (HRF) implemented in SPM8. The movement parameters obtained during the motion correction procedure were included as covariates of no interest into the model. In addition, artifact regressors were constructed if volumes showed artifacts (see above). If consecutive volumes were affected, they were combined into one regressor. To minimize ‘spill-over effects’ we included one volume immediately before and after the affected volumes into the regressor. The following functional contrasts were considered using t-tests on a voxel-by-voxel basis: (1) movement–rest, (2) rest–movement. Significance was assumed at a threshold of p < 0.05, whole brain FWE-corrected and resulting maps were superimposed onto the individual T2-data sets.

### Coil comparison

To further investigate the coil characteristics, we compared the dedicated neonatal coil with the standard adult head coil, using phantom and in vivo measurements. Because no ethical approval for direct in vivo coil comparisons was obtained, we calculated global and local (SMC) tSNR in the current study group and a previously acquired cohort [17].

### Phantom measurements

Phantom measurements were performed to compare tSNR values of the dedicated neonatal head coil and the standard 8-channel adult head coil previously used. An 11 cm diameter gel phantom was used to access the temporal SNR of both coils (3T-GEL-Phantom, Model Nr. 2360034, General Electric Company, Milwaukee, Wisconsin, USAI). Briefly, averaging across the acquired time series of 100 volumes, and dividing the obtained mean volumes voxel-wise by the standard deviation over time calculated the tSNR. The tSNR results between the studied coils are illustrated as colour map with overlaid ROI in **S1 Fig**.

### In vivo measurements

The tSNR ratio was calculated based on the acquired fMRI time series for two separate cohorts. The first data were taken from our previous study [17] acquired with the adult standard 8-channel head coil (Phillips, Best, NL) (cohort 1). These data were compared to the second actual dataset using the neonate specific head coil (cohort 2). Calculations were made by comparison of time series mean signal and standard deviation using the standard American College of Radiologists method, implemented using the yw_epi toolbox for Matlab (PNRC, Cincinnati). Briefly, noise was determined by acquiring data from non-signal producing regions in the corners of each image, calculating the average standard deviation within these voxels and dividing by the Rician distribution factor to determine whole image noise. Signal was determined regionally for the SMC by extracting mean signal over time for the whole functional sequence from regions of interest over both sensorimotor cortices (15mm diameter spheres positioned over the central sulcus at slice Z = +60 and summed across hemispheres) as well as averaged across the central 75% of the signal producing region (global signal). Each of these was divided by the whole image noise to give local and global tSNR respectively. The values for tSNR were statistically compared between groups using a 2 sampled t-Test implemented in SPSS Version 21 (IBM Corp, Arhok, NY).

### Results

Demographic data of study infants, structural MRI findings and routine cranial US examination results are given in **Table 1**.

**Table 1:** Demographic characterization of the study group.

Patient	Sex (m/f)	GA (weeks)	Birth weight (g)	IVH on day 7 cUS (Grade left/right)	PCA at MRI (weeks)	Structural MRI findings*
**NeoNat-01**	m	22 6/7	645	3/0	39 3/7	2
**NeoNat-03**	f	28 0/7	745	0	42 6/7	1
**NeoNat-06**	f	27 4/7	1120	0	37 6/7	1
**NeoNat-08**	f	27 4/7	1145	0	38 2/7	1
**NeoNat-09**	m	27 3/7	1280	0	39 1/7	2
**NeoNat-11**	m	25 2/7	715	3/2	38 5/7	1
**NeoNat-12**	f	25 2/7	400	0	40 3/7	1
**NeoNat-13**	m	25 4/7	800	2/2	41 0/7	1

Structural MRI findings in detail: Infant 01: cerebellar hemorrhage, basal ganglia lesion left, small corpus callosum; Infant 09: plexus cyst caudo-thalamic fossa, small periventricular white matter lesions, basal ganglia calcification.

*Description structural MRI changes on T1 and T2 sequences: 1 = normal, 2 = structural abnormalities. GA,gestational age at birth; IVH, intraventricular hemorrhage; cUS, cranial ultrasound; PCA, post-conceptional age at MRI examination.

The clinical neurodevelopmental assessments revealed no evidence of defined fine or gross motor dysfunction on neurodevelopmental follow-up at 21 month of age (median; 12–25 month range) in any of the reported neonates, as indicated in **Table 2**.

**Table 2:** Developmental Outcome according to the Bayley II Test and Neurological examination.

Patient	Age at Assessment (month)	MDI*	PDI*	DAM* (month)	Neurological Examination
**NeoNat-01**	25	72	63	20	1
**NeoNat-03**	20	100	104	21	1
**NeoNat-06**	24	112	117	26	1
**NeoNat-08**	22	112	98	25	1
**NeoNat-09**	22	112	91	25	1
**NeoNat-11**	13	82	69	10	1
**NeoNat-12**	16	88	66	14	1
**NeoNat-13**	12	87	70	11	1

* Bayley II Test; MDI, Mental Developmental Index; PDI, Psychomotor Developmental Index; DAM, Developmental Age of Motor Function. The listed assessment date refers to term equivalent age. Quality of Motor Function at Developmental testing: 1 = normal, 2 = CP. PDI < 80 in patients 01, 11, 12, due to low MDI result and poor general test performance (patients 01, 11) or not yet walking independently (patient 12).

### SNR comparison

To test the reliability of SNR improvement, tSNR analysis data of the clinical neonatal study from the n = 10 eligible data sets using the customized head coil (tSNR_custom_) were compared to n = 10 eligible data sets acquired with adult 8-Channel head coil (tSNR_adult_) as part of our previously published study [17]. In both datasets, tSNR was analyzed over the whole brain and over the SMC. Analysis of global tSNR revealed a significant improvement by 73% (tSNR_adult_ ± SD = 90.77 ± 22.6, tSNR_custom_ ± SD = 157.3 ± 47.5, p = 0.006), and a significant tSNR improvement in the sensorimotor cortex by 55% (tSNR_adult_ ± SD = 239.1 ± 64.1, tSNR_custom_ ± SD = 369.6 ± 111.8, p = 0.02) using the optimized neonatal coil (compared to the 8-channel adult head coil).

An 11cm diameter gel phantom was used to study the temporal SNR of customized head coil and the 8-Channel head coil used in our previous study. The observed improvement in tSNR for the neonatal coil was on average about 240% with a maximum about 700% close the single coil elements (see **S1 Fig**).

### fMRI experiment

**Fig 2:**
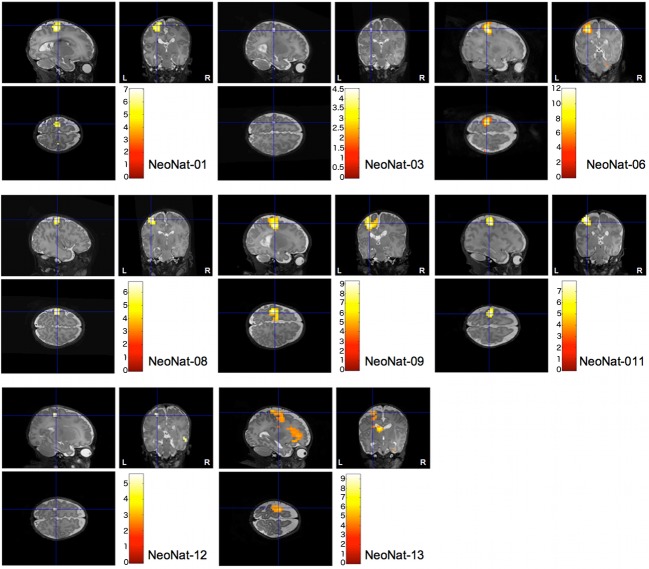
Individual fMRI activation patterns of all infants (N = 8). Individual fMRI results after stimulation of the right arm, statistical threshold at p < 0.05 (whole brain FWE-corrected). The resulting activation maps are superimposed onto individual normalized 3D-T2 volumes. The common pattern is that of a contralateral positive BOLD response in SMC. Only NeoNat-01 showed an additional small ipsilateral co-activation of the SMC (cluster size 2 voxels).

A significant positive BOLD response was elicited in all eight studied cases in the contralateral SMC (p < 0.05, FWE-corrected), representing a reproducible activation pattern across subjects (**Fig 2**). Ipsilateral SMC co-activation (p < 0.05, FWE-corrected) was seen in one neonate (NeoNat-01), even though the cluster extent was small (2 voxels). Additional activations were only rarely observed and were inconsistent (**Fig 2**, **Table 3**). In none of the cases was a significant deactivation detectable in the SMC.

**Table 3:** Summary of the fMRI results.

Patient	TE (msec)	Stimulation	fMRI response	BOLD	Cluster Size	T	Z
**NeoNat-01**	40	right arm	left SMC	positive	55	7.03	6.85
			right SMC	positive	2	4.69	4.63
**NeoNat-03**	35	right arm	left SMC	positive	2	4.49	4.44
**NeoNat-06**	40	right arm	left SMC	positive	93	12.04	Inf
	40	left arm	right SMC	positive	122	11.88	Inf
**NeoNat-08**	35	right arm	left SMC	positive	32	6.79	6.63
**NeoNat-09**	35	right arm	left SMC	positive	122	9.27	Inf
**NeoNat-11**	35	right arm	left SMC	positive	72	7.89	7.64
	40	right arm	left SMC	positive	26	6.54	6.4
**NeoNat-12**	40	right arm	left SMC	positive	6	5.09	4.88
**NeoNat-13**	40	right arm	left SMC	positive	97	5.60	5.53

Given are the cluster sizes, direction of the BOLD amplitude and maximum T / Z-scores reached within the activated cortical clusters. Because no common frame of reference is established for neonates, no coordinates are given. TE, echo time; SMC, sensorimotor cortex; Inf, infinite.

**Fig 3:**
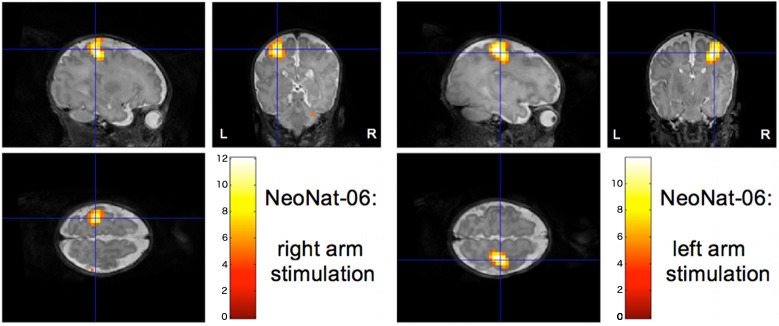
Activation of the sensorimotor cortex: Stimulation of the right arm and independent stimulation of the left arm. Individual activation pattern of two successive fMRI runs—superimposed onto the individual 3D-T2 data set (NeoNat-06): right-sided stimulation induces strict left-sided SMC activation and left-sided stimulation strict right-sided SMC activation (p < 0.05, whole brain FWE-corrected).

**Fig 4:**
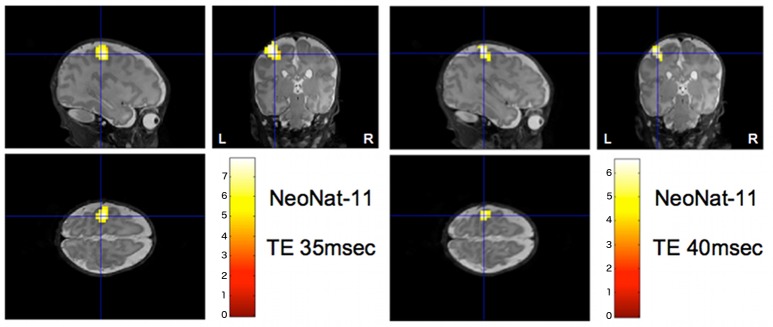
Activation of the sensorimotor cortex: Two successive fMRI runs with stimulation of the right arm. Individual activation pattern of two successive fMRI runs—superimposed onto the individual 3D-T2 data set (NeoNat-11); both with stimulation of the right arm, but using different echo times (35ms, 40 ms). Irrespective of the chosen echo time, a unilateral positive activation of the contralateral SMC cortex is detected (p < 0.05, whole brain FWE-corrected).

Additionally, the cases with two successful consecutive fMRI runs both revealed conclusive activation patterns: In NeoNat-06, the stimulation of the left arm elicited a focal activation of the right SMC, while the stimulation of the right arm elicited a focal activation of the left SMC (**Fig 3**); In NeoNat-11, the two runs with different echo times revealed in both cases a left sided SMC activation (**Fig 4**).

## Discussion

To improve the detection and reproducibility of neonatal sensorimotor mapping, we employed a purpose built neonatal head coil that was specifically developed and optimized to the geometry and size of the neonatal head after appropriate safety testing procedures (Philips, Best, NL). Compared to a standard 8-channel adult head coil used previously by our group [17], phantom studies showed a mean 2.4 fold increase in SNR. Indirect comparisons of the two head coils in independent neonates, revealed a significant improvement in global (73%) and local (55% in sensorimotor cortex ROI) tSNR of the neonatal head coil. Upon unilateral sensorimotor stimulation, significant positive BOLD responses could be identified in contralateral SMC of all studied (N = 8) infants (10 total data sets). This confirmed our hypothesis that improving quality of acquired data would lead to a robust, contralateral SMC response to stimulation. This was consistent with recent fMRI studies using improved analytical methods that account for age-dependent HRF-modelling [21, 24]. Compared to previous neonatal sensorimotor fMRI studies employing adult coils, the reliable detection of evoked BOLD responses with robust statistical control (i.e. p < 0.05, FWE-corrected) is an important methodological improvement. Previous neonatal fMRI studies targeting the SMC have not always demonstrated use of robust statistical control [17–19] and, therefore, previously reported data should be considered with care due to intrinsically high rates of false-positives. Therefore, the goal of providing an early functional biomarker that is informative for individual at-risk assessments at this point of brain development is unlikely to be met by previously described approaches.

In each successfully analyzed functional dataset (i.e. not contaminated by gross task-associated head motion artifacts hampering meaningful data analysis), we identified positive BOLD responses almost exclusively in the contralateral SMC. Our single subject findings differ not only to most previous studies by the improved statistical robustness, but also with regard to the consistent direction and laterality of evoked BOLD responses to unilateral sensorimotor stimulation. BOLD signals in previous somatosensory stimulation studies ranged from predominantly positive [21], mixed positive /negative BOLD signals [19] to predominantly negative responses [17]. A recent study that has reported comparable findings to ours [21] applied cluster-wise correction, providing more robust statistical control than many previous studies, although less strict than those employed in our current work. Those authors identified mainly positive contralateral BOLD responses to unilateral stimulation induced by single hand extension movements that were generated by an automated stimulation device [21, 22]. While the stimulation events were arranged in a block-type fashion, similar to our study, it is important to point out that the performed statistical analysis was based on flexible individual modelling of the hemodynamic responses to account for potential deviations of the neonatal hemodynamic response from the response function observed in the adult brain. This approach—combined with carefully designed artifact removal—did improve the robustness and statistical significance of the presented fMRI data [21, 22], rendering the reported results more reliable than all previous findings reported so far, including our own initial work [17].

Here, we show that sensorimotor fMRI acquired with an optimized neonatal head coil allows reliable and robust analysis. The higher tSNR afforded by the custom neonatal head coil allowed detection of positive findings despite application of a robust statistical threshold set at p < 0.05, FWE-corrected (applied at the single-subject level). All examined preterm infants showed a lateralized positive BOLD response and a normal sensorimotor development on standardized neurodevelopmental testing up to the second year of life. These findings support recent evidence for an early functional lateralization of the sensorimotor system, despite the known bilateral anatomical architecture of the corticospinal system [31]. The observed lateralization of evoked SMC responses implies a considerable degree of functional lateralization already developed at this early postnatal stage, as also suggested by Arichi and colleagues [20, 21].

It is important to consider the current findings in the context of early development of human corticospinal connections: Functional monosynaptic corticospinal projections to motor neurons and spinal interneurons are established prenatally during the final trimester of pregnancy. Perinatally, there is more extensive innervation of spinal neurons early myelination of corticospinal axons [29], which can be demonstrated in the posterior limb of the internal capsule at TEA. Later, within the first two postnatal years, these bilateral corticospinal connections undergo lateralization through successive elimination of most ipsilateral connections [29, 31, 32]. Maturational changes of cortico-cortical (in particular transcallosal) connections during prenatal / postnatal periods are also important to consider here: experimental work in rhesus monkeys showed a progressive increase in the total number of callosal axons from mid-gestation through birth, before progressive maturational refinement takes place postnatally via callosal axon elimination [45]. In the human brain, unmyelinated callosal fibers can be identified as early as 28 weeks PCA by means of DTI measurements [46]. From thereon, maturation of callosal connections is known to continue over a timescale of years, as shown by DTI anisotropy measurements [47]. Specific knowledge regarding maturation of intrahemispheric (non-callosal) cortico-cortical connections is sparse [48], but cortical projection neurons initially have widespread distributions and then become more restricted during development through elimination of functionally inappropriate axon segments [49].

Despite the intrinsic anatomical bilaterality of corticospinal axons and the existence of transcallosal connections in neonates, the work by Arichi et al. [21] and our presented data provide new and very interesting evidence of a distinct functional lateralization of the postnatal sensorimotor system. In both studies, unilateral passive movements elicited preferential contralateral SMC activation, comparable to findings in older children [16] and adults [26–28]. Importantly, our findings should not be interpreted as evidence that unilateral sensorimotor simulation in neonates is processed exclusively via crossed corticospinal connections; rather, they imply that already at the early postnatal stage, a considerable degree of functional lateralization has developed, preferentially involving lateralized sensorimotor circuits. This highlights the value of functional, compared to purely structural, imaging of the neonatal motor system as a potential biomarker of future motor development.

The reported SMC activation to the unilateral passive flexion/extension movements of the upper extremity has important implications, as the contralateral positive BOLD response may serve as a simple functional viability marker in neonate at-risk states. For example, from a clinical perspective, a relevant finding might be a reduced / impaired contralateral SMC activation (potentially combined with compensatory enhanced ipsilateral activation), linked with structural lesions affecting the CST. Based on the current findings, unilateral hemispheric lesions might be expected to induce abnormal hemispheric BOLD responses, e.g. extrapolating from case NeoNat-06 where right-sided stimulation revealed strict left-sided SMC activation and vice versa left-sided stimulation strict right-sided SMC activation. We do recognize that translation of such functional findings into clinical diagnosis tools requires substantial further investigation but improved robustness and reproducibility of neonatal fMRI is an important prerequisite for applying this method as a viable candidate biomarker assessing early sensorimotor development in neonates. In the subjects where it was possible to complete consecutive fMRI runs, we observed remarkable consistency of the evoked SMC activation patterns.

It is interesting that one of our cases (NeoNat-01) showed evidence for additional ipsilateral activation of the SMC. A recent longitudinal fMRI study applying serial task fMRI (passive and spontaneous right wrist movements) and resting state connectivity in patients from 30–43 weeks PCA has provided first functional insight into the development of inter-hemispheric transcallosal connectivity and describes the increasing integration of ipsilateral hemisphere and secondary motor areas during this period of brain development [24]. A bilateral activation pattern following unilateral task performance might thus reflect an advanced developmental stage of the sensorimotor system [24]. In our study, this hypothesis cannot be directly tested, considering the observational character and the lack of data with clear pathology affecting the CST. Neurophysiological evidence arising from experimental work [32, 50, 51] and clinical observations in patients showing brain re-organization following unilateral hemispheric lesions [52] describe activity-driven development of CST functionality [53] whereby ipsilateral CST projections contribute to “reorganization” of limb function following perinatal insults [31, 54]. Further longitudinal fMRI studies employing robust methods as described may allow definite conclusions to be drawn on the relationship of activation patterns and clinical outcomes in this early stage of brain development.

Despite the reported methodological improvements of this study, several aspects need to be considered critically: Firstly, although the current study has applied a similar stimulation paradigm and MRI setting as described in our previous study [17], the SNR comparison of the different coils was performed using data sets from consecutive studies involving different individuals. The conditions of the study approval did not allow direct comparisons of the neonatal head coil and the standard adult head coil in the same neonate. Nevertheless, this explanatory analysis revealed a much higher incidence of significant contralateral positive BOLD responses with the dedicated neonatal head coil (100%), compared to the standard adult head coil (10%). Secondly, our study was exclusively performed in preterm neonates, scanned at TEA, presenting with no evidence of gross abnormal motor function on later neurodevelopmental follow-up at 18–24 months of age. Whilst we do not have direct evidence of this pattern of activity being present in healthy term-born infants, there is little reason to suspect that such results would differ substantially from the lateralized activation pattern described here and in recent research [21, 24]. Thirdly, the full clinical utility of the presented procedures can only be estimated when investigations in preterm infants reveal specific alterations of evoked stimulation patterns associated with adverse individual neurodevelopmental outcomes.

## Conclusions

An optimized head coil significantly improves signal detection in neonatal fMRI, thus improving reliability and robustness of fMRI in neonates. Employing these methods, our study provides evidence for robust lateralized cortical activation in response to unilateral sensorimotor stimulation in a cohort of preterm infants with subsequently documented normal motor development. Optimized neonatal fMRI might provide additional functional insight in physiological and pathophysiological brain development, adding important information to structural MRI studies.

## Supporting Information

S1 File Surface File of Neonatal Head Model. Depicted is the file used for constructing the neonatal head model based on 23 T1-weighted MRI datasets of term-born babies scanned at the age of 6 months.
(STL)

S1 Fig **SNR Comparison of Neonatal 8-channel Head coil (A) to the commercial 8-channel Adult Head Coil (B).** SNR comparisons between sagittal images obtained from head phantom study using the neonatal 8-channel head coil (row A) and the 8-channel adult head coil (row B). The images present the enhanced SNR results for the phantom in the neonatal coil. Color map with overlaid ROI.
(TIFF)
