# Supplementary figures and images for: Functional Laterality of Task-Evoked Activation in Sensorimotor Cortex of Preterm Infants: An Optimized 3 T fMRI Study Employing a Customized Neonatal Head Coil
Source: PLoS One. 2017 Jan 11;12(1):e0169392. doi: 10.1371/journal.pone.0169392 (PMC5226735; doi:10.1371/journal.pone.0169392)

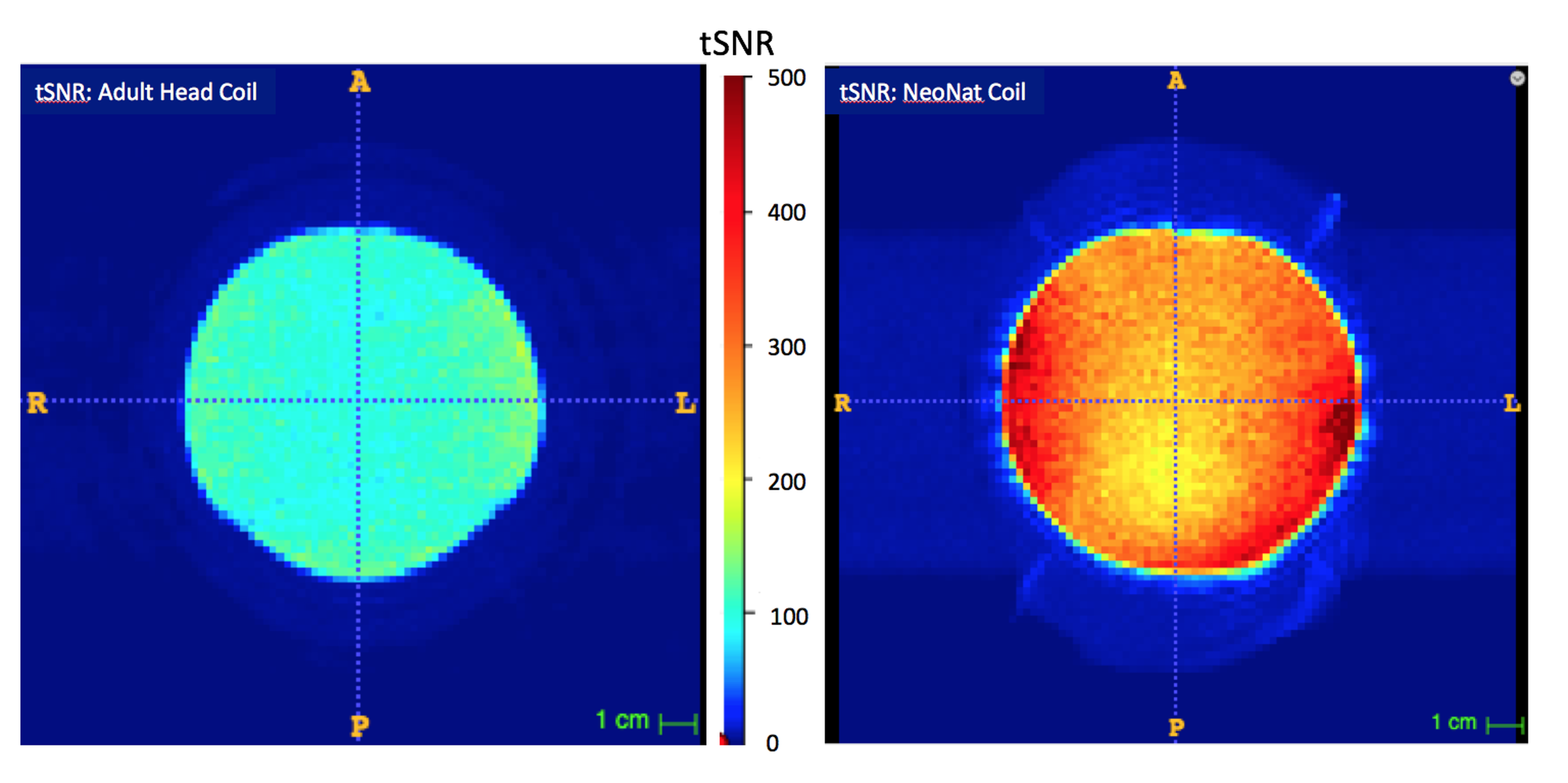

Supplement: S1 Fig — SNR Comparison of Neonatal 8-channel Head coil (A) to the commercial 8-channel Adult Head Coil (B). SNR comparisons between sagittal images obtained from head phantom study using the neonatal 8-channel head coil (row A) and the 8-channel adult head coil (row B). The images present the enhanced SNR results for the phantom in the neonatal coil. Color map with overlaid ROI. (TIFF) [file pone.0169392.s002.tiff]
